# Ultra-processed food consumption, dietary behaviors, and marketing exposure among school adolescents in urban Ethiopia: a qualitative study

**DOI:** 10.1186/s40795-026-01401-5

**Published:** 2026-06-15

**Authors:** Kefyalew Taye Belete, Simon Nitter Dankel, Tafese Bosha, Gudina Egata

**Affiliations:** 1 School of Nutrition, Food Science and Technology, College of Agriculture, Hawassa University, Hawassa, Ethiopia; 2 Department of Public Health, College of Health Sciences and Referral Hospital, Ambo university, Ambo, Ethiopia; 3 Centre for Nutrition, Department of Clinical Science, University of Bergen, Bergen, N-5020 Norway; 4 Department of Public Health Nutrition and Dietetics, School of Public Health, College of Health Sciences, Addis Ababa University, Addis Ababa, Ethiopia

**Keywords:** Adolescents, UPFs, Dietary behavior, Food marketing, Ethiopia

## Abstract

**Background:**

Adolescence is a critical period for establishing lifelong dietary habits. The growing availability and marketing of ultra-processed foods (UPFs) pose increasing public health nutrition concerns, particularly in rapidly urbanizing settings. However, qualitative evidence on how adolescents in urban Ethiopia engage with UPFs remains limited. This study explored UPF-related dietary behaviors, consumption patterns, and exposure to food marketing among school adolescents in Adama Town, Ethiopia.

**Methods:**

A qualitative study was conducted using 15 in-depth interviews with purposively selected secondary-school adolescents aged 13–19 years to ensure variation by age, sex, and school type. Interviews were conducted in Afan Oromo, audio-recorded, transcribed, translated into English, and analyzed thematically using Braun and Clarke’s framework. Trustworthiness was enhanced through peer debriefing, reflexivity, and audit trails.

**Results:**

Seven interrelated themes described adolescents’ dietary behaviors, consumption patterns, marketing exposure, and recommended responses related to UPFs. Adolescents expressed ambivalent views, describing UPFs as hygienic, quality-assured, affordable, and timesaving, while also recognizing concerns related to artificial ingredients, high sugar and fat content, and long-term health effects. Consumption ranged from frequent intake of packaged snacks, sugary drinks, and some commercially prepared staple foods to occasional fast-food consumption, shaped by taste, availability, affordability, situational need, and social influences from family and peers.

Participants identified perceived benefits, including quick energy, weight gain, durability, and trust in the safety of commercially produced foods, alongside recognized risks such as dental problems, obesity, chronic diseases, addiction, and exposure to expired or contaminated products. Exposure to food marketing through mass media, packaging, and branding increased awareness and desire for UPFs, although some adolescents expressed skepticism toward misleading promotions and held mixed views on marketing restrictions. Despite consumption, many adolescents demonstrated reflective awareness and recommended moderation, preference for natural or homemade foods, checking expiry dates, and stronger regulation and consumer education to reduce harms.

**Conclusion:**

School adolescents in urban Ethiopia navigate a complex interplay of taste, convenience, affordability, social influence, and health awareness in their engagement with UPFs. Public health nutrition interventions should address both individual dietary behaviors and the broader food environment, including exposure to food marketing, to promote healthier dietary choices among adolescents.

**Supplementary Information:**

The online version contains supplementary material available at 10.1186/s40795-026-01401-5.

## Introduction

Overweight and obesity among children and adolescents have emerged as global public health challenges. Current estimates suggest that one in four children and adolescents worldwide live with overweight or obesity, a prevalence that continues to rise [1]. Adolescent obesity is particularly concerning because it often persists into adulthood, increasing the risk of non-communicable diseases (NCDs) and premature mortality [2]. In Sub-Saharan Africa (SSA), the urgency to address adolescent obesity is amplified by the region’s enduring double burden of malnutrition, where undernutrition coexists with a rapidly growing epidemic of overweight and obesity [3].

One of the most prominent drivers of this nutritional transition is the increased consumption of ultra-processed foods (UPFs), which have increasingly replaced traditional food cultures across the world during the last decades. UPFs, as defined by the NOVA food classification system, refer to NOVA group 4 foods, which are defined as industrial formulations of ingredients with little or no whole food content, are typically high in added sugars, salt, and processed fats, while low in fiber and micronutrients [4]. Evidence consistently links high UPFs intake to poor dietary quality, obesity, hypertension, metabolic syndrome, and even certain cancers [5, 6]. Adolescents are disproportionately exposed to UPFs due to their affordability, convenience, hedonic appeal, and targeted marketing [7]. Studies from diverse settings including Brazil, Canada, Belgium, and Australia indicate that UPFs contribute between one-third and two-thirds of adolescents’ daily caloric intake [8–10].

In many low- and middle-income countries (LMICs), including Ethiopia, rapid urbanization is reshaping food environments. Urban living is associated with greater access to supermarkets, convenience stores, and street vendors offering UPFs, often displacing traditional dietary patterns [11, 12]. With global UPF markets becoming increasingly saturated in high-income countries, transnational food corporations have aggressively expanded into LMICs, employing sophisticated marketing strategies that target youth populations [13]. As a result, adolescents in LMICs are particularly vulnerable to unhealthy dietary transitions, where higher energy intake from UPFs is accompanied by less rather than greater dietary diversity [14].

Traditionally, diets among Ethiopian adolescents are centered on minimally processed, home-prepared foods such as injera (a fermented flatbread made from teff), legumes, vegetables, and locally prepared stews (wot/shiro) [15]. These diets are generally characterized by low levels of industrial processing and limited use of packaged food products [16]. However, rapid urbanization is contributing to shifts toward more commercially prepared and packaged food consumption [17].

In Ethiopia, NCDs already account for an estimated 39% of all deaths, with one in five occurring prematurely among younger age groups [18]. Urban centers appear disproportionately affected, with recent evidence from Addis Ababa reporting prevalence of NCDs as high as 63.2% and suggesting that more than half of such deaths could have been prevented through modification of lifestyle-related risk factors [19]. Although adolescent obesity remains moderate in Ethiopia (10%) [20, 21], the trajectory observed in other LMICs suggests a likely escalation if early prevention measures are not implemented. Despite the growing concern, Ethiopia currently lacks policies regulating UPF marketing, especially in and around schools, allowing a continued and growing replacement of traditional diet by UPFs [22].

The role of psychosocial, environmental, and marketing factors in shaping adolescents’ dietary behaviors is well documented globally. Taste, convenience, affordability, and social norms remain dominant drivers of UPF consumption [23]. Likewise, school and neighborhood food environments strongly influence adolescents’ exposure to and access to packaged and commercially processed foods [24]. In many urban African settings, advertising around schools and public spaces predominantly promotes sugar-sweetened beverages, packaged snacks, and fast foods, reinforcing preferences for energy-dense, nutrient-poor items [25]. While similar trends are emerging in Ethiopia, qualitative evidence capturing how adolescents themselves understand these products and how they interpret the marketing surrounding them remains limited.

This gap is particularly important because adolescence is a crucial developmental stage for forming lifelong dietary habits [26]. Moreover, adolescents have heightened sensitivity to food marketing [27]. In Ethiopia, adolescents often identify specific UPFs such as biscuits, chips, sugary drinks, pizza, and burgers, yet awareness of the technical term “ultra-processed foods” or the NOVA classification remains unclear and is likely limited, as this has not been directly assessed [28]. Understanding how Ethiopian urban adolescents perceive UPFs, what motivates their consumption, and how they experience food marketing is therefore essential. This qualitative study examines these issues in Adama Town, generating contextual insights into how autonomy, peer influence, affordability, convenience, and routine exposure to food advertising interact to shape adolescents’ dietary choices. These findings can help guide adolescent-focused interventions and inform national nutrition strategies aimed at reducing future NCD risks.

## Methods

### Study design

A qualitative design using thematic analysis was employed to explore adolescents’ perceptions, behaviors, and exposure to UPF marketing using an In-depth interview (IDIs) technique. This approach was selected to enable exploration of adolescents’ individual perceptions and experiences in a confidential setting. This approach was preferred over focus group discussions to minimize peer influence and social desirability bias, and to allow participants to freely express personal views on dietary behaviors and food choices.

### Study setting and participants

The study was conducted among secondary school adolescents (13–19 years) in both public and private schools in Adama City Administration, located in the Oromia Regional State of central Ethiopia. The city had an estimated population of 504,345 in 2024 based on projections from the 2007 national population and housing census [29] and is a rapidly urbanizing commercial and transportation hub characterized by diverse socioeconomic conditions and evolving food environments. Schools were selected to represent diverse socioeconomic backgrounds. Within each school, participants were purposively recruited to ensure variation in age, sex, and school type. Recruitment was facilitated through school principals, teachers, and shift leaders (teacher who responsible for coordinating school activities within a given shift) who announced the study using flyers. Adolescents who expressed interest were approached individually and provided with further information. A total of 15 IDIs were conducted, with sample size guided by thematic saturation.

Adama Town is experiencing ongoing nutrition transition, characterized by increasing availability of commercially processed and packaged foods alongside persistent undernutrition and micronutrient deficiencies [30, 31]. Like many urban settings in Ethiopia, adolescents are exposed to a mixed food environment, including traditional home-prepared foods and a growing number of street vendors, small shops, and supermarkets selling packaged products [32].

School contexts also differ, with private schools often attended by adolescents from relatively higher socioeconomic backgrounds and with greater access to discretionary spending, while public school students may have more limited resources [28]. Formal school meal programs are not consistently implemented in this setting, and most adolescents obtain food from home or purchase it from vendors in and around school environments.

### Ethical considerations

Ethical approval for the study was obtained from the appropriate institutional review board. Written informed consent was obtained from participants aged 18 years and above, and assent with parental consent was secured for minors.

### Data collection

Data were collected between February and March 2025. Semi-structured, face-to-face IDIs were conducted using an interview guide informed by the study objectives and relevant literature. The guide included 17 open-ended questions exploring adolescents’ perceptions of UPFs, perceived benefits and risks, motivations and barriers to consumption, and exposure to UPF marketing. Furthermore, sociodemographic characteristics, including age, sex, and school type, were collected from all participants.

The interview guide was piloted with a small number of adolescents outside the study sample to assess clarity, and flow. Minor revisions were made to improve question wording and sequencing. The final interview guide is provided as a supplementary appendix.

At the beginning of each interview, participants were asked whether they had previously heard the terms *“processed foods”* or *“ultra-processed foods”* and to explain their own understanding. This allowed us to document their baseline knowledge. To avoid influencing responses, no definitions or examples were provided by the interviewer; participants relied on their own conceptualizations, which commonly included familiar packaged or commercially prepared items such as biscuits, chips, sugary drinks, pizza, or burgers. Throughout this study, references to “industrial,” “processed,” or “packaged” foods reflect participants’ everyday understandings and descriptions rather than formal nutrition classifications. These lay descriptions may overlap with, but do not necessarily correspond directly to ultra-processed foods as defined by the NOVA classification.

Interviews were conducted by FDM, a trained Master of Public Health (MPH) graduate who had no prior relationship with the participants. Interviews were conducted during school days at times arranged in coordination with school staff, typically outside of regular class hours to avoid disruption of academic activities. All interviews took place in private spaces within school compounds to ensure confidentiality and comfort, and teachers were not present. Interviews were conducted in Afan Oromo, and were audio-recorded with participants’ consent. Field notes were taken to capture contextual details and non-verbal cues that supported interpretation during analysis.

### Data analysis

Interviews were conducted in Afan Oromo, audio-recorded, and later transcribed verbatim. Transcripts were translated into English by bilingual researchers familiar with both languages and the study context. A second bilingual reviewer cross-checked translations against original audio recordings to ensure accuracy and preserve meaning equivalence. Data were imported into NVivo 12 for management and systematic coding. Thematic analysis followed Braun and Clarke’s six-phase framework: familiarization, initial coding, theme development, review, definition, and reporting [33]. Coding was conducted using a primarily deductive approach, guided by the study objectives and interview domains. At the same time, the analysis remained flexible to allow new themes and insights to emerge inductively from the data. Coding was conducted by the first author (KTB). Constant comparison across transcripts ensured consistency and rigor, and findings were presented thematically with illustrative quotes.

### Trustworthiness

Credibility of the findings was strengthened through iterative engagement with the data, including repeated reading of transcripts, peer debriefing with qualitative research colleagues, and regular team discussions to refine emerging themes. Dependability and confirmability were supported by maintaining a detailed audit trail, including coding decisions, analytic memos, and documentation of theme development. This process was led by the primary researcher and periodically reviewed with a senior qualitative mentor to ensure consistency and transparency. Reflexivity was addressed through ongoing journaling by the primary researcher, reflecting on how their background in nutrition and public health may have influenced data interpretation.

## Result

### Sample characteristics

Between February-March 2025, fifteen in-depth interviews (lasted 35–50 min, mean time 42 min) were conducted with adolescents aged 13–19 years from public and private secondary schools in Adama City, Ethiopia. Results are presented thematically across seven interrelated themes describing adolescents’ understanding, perceptions, consumption behaviors, motivations, and responses related to ultra-processed foods. Demographic details are presented in Table 1.

**Table 1:** Socio-demographic characteristics of participants

Variable	Category	Number	Percent
Sex	Male	9	60%
	Female	6	40%
Age (years)	Mean (years)	16.5 years	
School type	Public	8	54%
	Private	7	46%

### Overview of themes

Thematic analysis identified seven overarching themes that together illustrate adolescents’ perceptions, intentions, behaviors, and responses regarding UPF consumption in Adama Town. These include Perceptions and Understanding of UPFs, intention to consume, consumption behaviors, perceived benefits, perceived risks, marketing exposure and effects, and recommendations (supplementary Table 2).

Participants described UPFs as clean, quality-assured, and timesaving, while also noting their affordability and convenience. At the same time, they acknowledged both nutritional benefits and health risks, raising concerns about contamination, expiry dates, and misleading advertising.

Taste, convenience, and affordability were strong motivators of UPF consumption, but many participants demonstrated awareness of potential harm and voiced recommendations such as limiting intake, preferring natural or homemade foods, checking expiry dates, and strengthening regulation and education. This thematic structure reflects the interplay between personal preferences, social influences, and environmental factors in shaping adolescent UPF consumption.

### Theme 1: perceptions and understanding of ultra-processed foods

Adolescents’ perceptions of UPFs were closely linked to their underlying understanding and interpretation of these foods. Many participants conceptualized UPFs based on visible characteristics such as packaging, expiry dates, and perceived hygiene, rather than levels of processing. This indicates that their understanding does not necessarily align with the NOVA classification.

Furthermore, they expressed diverse and at times ambivalent views of UPFs, reflecting both attraction to their convenience and concern about their risks. Some described commercially packaged foods as safe and hygienic, emphasizing expiry dates, intact packaging, or repeated testing as markers of quality. For example, one participant explained, *“I don’t think these foods can cause any problems if they are not expired and their covers are not torn”* (IDI 1, Male, 14 years old), while another pointed to bottled water as a product that undergoes repeated checks before release (IDI 12, male, 19 years old).

At the same time, UPFs were valued for their affordability and time-saving qualities, with participants noting how easily they fit into daily routines. As one adolescent put it, *“Industrial food is positive because it saves us time…we can get it whenever we want”* (IDI 7, female, 17 years old). For others, the low cost itself was a motivator for frequent consumption (IDI 14, male, 17 years old).

Yet concerns were also voiced about artificial additives, excessive fat and sugar, and long-term illness. One participant warned that *“biscuits and other supplements contain more chemicals than natural substances and can cause various harms”* (IDI 2, male, 18 years old), while another linked frequent consumption to obesity, diabetes, and hypertension (IDI 13, male, 19 years old).

For many, these apparently conflicting perspectives coexisted. The same adolescent who praised UPFs for their convenience also expressed caution: *“Something that has advantages has disadvantages… it saves us time*,* but it is not good to take it continuously”* (IDI 7, Female, 17 years old). Such reflections suggest that adolescents do not hold fixed views but rather negotiate between the practical benefits of UPFs and their potential health risks, often arriving at moderation as a compromise.

### Theme 2: intention to consume UPFs

Adolescents often framed UPFs consumption as a personal choice: *“I use these foods of my own free will*,* not under pressure from others”* (IDI 2, male, 18 years old). Similarly, (IDI 3, female, 16 years old) emphasized autonomy in selecting packaged foods. At the same time, family routines shaped choices, with one explaining: *“If we go with children*,* we will have to do what they say because it depends on their choices”* (IDI 2, male, 18 years old). Peers also influenced decisions: *“If my friends who used it before invite me*,* I also buy it”* (IDI 5, Female, 15 years old).

Situational need was another factor some relied on UPFs when no food was available at home: *“If there is no food at home*,* I buy biscuits or bread from the shop nearby”* (IDI 4, male, 14 years old). Affordability further enabled uptake: *“They are affordable compared to other foods sold in restaurants”* (IDI 8, female 17 years old). Furthermore, taste preference and perceived convenience also shaped adolescents’ intention to consume these foods. *“The reason why they choose it is because it tastes good”* (IDI 2, male 18 years old). *“We buy biscuits and drinks because they are sold at reasonable prices and can be obtained when needed”* (IDI 1, male 14 years old).

### Theme 3: consumption behaviors

Reported consumption ranged from daily to occasional. Popular items included biscuits, chips, and sugary drinks: *“Among the covered foods*,* the one we buy and eat is biscuits*,* because they are easily available in many places”* (IDI 1, male 14). Others highlighted fast foods like pizza and burgers (IDI 10, female, 15 years old). Participants also reported consuming foods such as bread, pasta, and macaroni; however, these items may vary in their level of processing depending on preparation (homemade vs. commercially produced), and therefore may not all be classified as UPFs: *“My favorite foods… are bread*,* sambuusa*,* macaroni*,* but I prefer bread most of all”* (IDI 1, male 14 years old).

Consumption patterns varied considerably between participants. Some adolescents expressed a preference for fruits and vegetables because of their freshness and perceived health benefits: *“My favorite food that I like most are fruits. Because fruits are mostly fresh*,* they are available around us*,* and we know their benefits. … I prefer them because they are useful.”* (IDI 2, male, 18 years old). In contrast, others described frequent consumption of UPFs, whereas some emphasized moderation, limiting intake to only a few occasions per week or month: *“Occasionally I use pizza and burgers etc. once a month.”* (IDI 14, male, 17 years old). Overall, these consumption patterns were shaped by taste, availability, affordability, and situational convenience. Some adolescents also justified their choices based on perceived health or energy benefits, particularly when foods were considered safe or not expired.

### Theme 4: perceived benefits

Adolescents often viewed UPFs as nutritious and energy-giving, though the sources of these beliefs were unclear and likely influenced by marketing, packaging, or social norms: *“Eating these industrially produced foods is good for our health and helps us to gain weight”* (IDI 3, female, 16 years old). Others noted their role in providing quick energy (IDI 11, male 17 years old). Trust in industrial safety and durability was also highlighted: *“Produced after being treated with chemicals and tested by experts*,* so they last longer and are of high quality”* (IDI 5, female, 15 years old), with IDI 6 (male 17 years old) adding confidence that “*there is no problem with the products produced in the industry.*” Some also emphasized affordability (IDI 1 male 14 years old, IDI 8 female 17 years old) and broader social value, such as strengthening friendships through shared meals (IDI 5, female 15 years old).

### Theme 5: perceived risks

Despite recognizing benefits, adolescents voiced significant concerns about the risks of UPFs, particularly dental problems, obesity, and chronic disease. As one explained: *“Eating too much sweet foods can damage our teeth… and addictive products can damage our lungs”* (IDI 2 male 18 years old). Others linked UPFs to long-term illnesses: *“Industrially produced foods have both harms and little benefits. The side effects can lead to diabetes*,* cancer and the like”* (IDI 14, male 17 years old).

Participants also worried about expired or contaminated products: *“Before taking these foods*,* you must verify who made them and ensure that they are not expired”* (IDI 1, male 14 years old). Some further described addictive tendencies, suggesting UPFs could foster dependency. These narratives reflect an ambivalence between trust in industrial safety and recognition of hidden harms.

### Theme 6: marketing exposure and effects

Advertising strongly influenced adolescents’ choices. Some described being drawn to try foods after seeing them in media: *“When these foods are advertised in the media*,* they stimulate our desire to buy and eat them”* (IDI 2, male 18 years old). Some admitted feeling pressured to consume advertised products (IDI 14, male 17 years old). Packaging and branding further reinforced desirability: *“Advertising plays an important role in reaching the consumer. It will make the public aware*,* make them aware of the types of food available*,* and make them know their place”* (IDI 9 male 15 years old). *“The industrially prepared foods should be advertivsed… so that consumers can distinguish the right product from the wrong one.”* (IDI 10 female 15 years old). Yet some expressed skepticism about exaggeration: *“I think it would be harmful if they broadcast something that is not true”* (IDI 1 male 14 years old). Participants expressed mixed views on advertising restrictions, with both supportive and opposing perspectives (IDI 9 male 15 years old, IDI 10, female 15 years old): *“I don’t feel anything about being banned from advertising*,* but I feel good if healthy foods are advertised.”* (IDI 7 female 17 years old).

*“I don’t feel good about advertising of unhealthy foods*,* but healthy food should be advertised.”* (IDI 15 female 16 years old).

### Theme 7: recommendations

Adolescents offered a range of strategies to reduce risks. They also emphasized practical strategies such as checking expiry dates and verifying product safety. Moderation was another common recommendation: *“These foods taste good… but can give us great benefits if we use only the amount*,* we need without too much”* (IDI 6, male 18 years old). Others promoted natural foods: *“It is better if we use natural ingredients around us”* (IDI 2, male 18 years old), with stronger warnings from IDI 14, male 17 years old: *“My advice is not to eat*,* it is useless.*

Finally, Participants emphasized the need for affordable access to healthier food options, stronger enforcement of food quality and safety standards (e.g., monitoring expiry dates and product quality), and improved consumer awareness regarding the health effects of frequent UPF consumption. As one participant noted, “*companies should provide healthy foods at reasonable cost so even the poor can buy and use them*” (IDI 5, female 15 years old), highlighting concerns about affordability and access. Others emphasized the importance of quality control: “*Industries… should produce in accordance with the standards*” (IDI 8, female 17 years old). Participants also reflected on the need for awareness and moderation, with one stating that “*industrially produced foods… [are] harmful if used continuously*,* unless used occasionally*…” (IDI 11, male 17 years old), suggesting a need for better understanding of appropriate consumption patterns.

## Discussion

This study explored adolescents’ understanding and perceptions, intentions, and behaviors regarding UPF consumption in Adama Town, revealing a nuanced interplay between personal preferences, social influences, environmental access, and marketing exposure.

Adolescents often expressed mixed views about UPFs. On one hand, they appreciated their convenience, affordability, and the sense of safety suggested by packaging and expiry labels. On the other, they acknowledged possible harms, including obesity, chronic illness, and dental problems. Such contradictions are consistent with international findings that young people tend to weigh the immediate enjoyment of UPFs against emerging concerns about health [34, 35]. This tension suggests that effective interventions must address not only awareness but also the structural conditions that shape food choices.

An important finding was that adolescents described commercially packaged or industrially produced foods using their own everyday understandings, which did not always correspond to the scientific definition of UPFs under the NOVA classification. Participants commonly interpreted these foods based on visible characteristics such as packaging, expiry dates, and perceived hygiene, rather than the extent and purpose of industrial processing. Many therefore associate commercially packaged or industrially produced foods with safety or quality. Similar observations have been reported in other settings and suggest that public health communication should build on adolescents’ existing understandings while providing clearer explanations about food processing and its potential health implications [36, 37].

The tendency to justify UPF intake because they are quick, inexpensive, or the most accessible option is especially relevant in rapidly urbanizing LMICs [38]. Research from Ethiopia and other similar settings shows that nutrition transitions and changing school food environments increase adolescents’ exposure to cheap, energy-dense products [22]. In our study, UPFs served both as snacks and as convenient substitutes for staple meals, demonstrating how environmental pressures such as cost, time, and availability interact with individual choices. These findings support calls for strategies that modify food environments alongside nutritional education.

Our results also echo conceptual work describing UPFs as distinct not just for their nutrient profile, but for their industrial formulation and presentation. The NOVA classification emphasizes how processing, additives, and branding influence consumer trust. Adolescents in this study frequently equated sealed packaging and expiry dates with safety, reflecting the social and technical signals that normalize UPFs [39]. However, the underlying sources of these beliefs were not systematically explored in this study.

Intentions around consumption were shaped by a mix of autonomy, family practices, peer influence, and economic realities. While many adolescents framed their eating as independent decision-making, their accounts showed clear influence from household routines, peer groups, and the foods readily available to them. In some cases, younger siblings’ preferences also appeared to influence food choices, suggesting a form of “pester power” that may extend beyond parents to older adolescents. Such dynamics fit ecological models of food choice [40]. In practice, UPFs often functioned as “default” options chosen when healthier alternatives were either scarce or unaffordable.

Behaviorally, frequent consumption of snacks, sugary beverages, and processed staples was reported, with taste and sensory appeal being decisive factors. This pattern parallels other LMIC contexts where the pleasure of flavor often outweighs health concerns [41]. Yet, the fact that many adolescents already recognized potential risks suggests an opening for interventions that build on existing awareness, rather than assuming a blank slate.

A key insight is that adolescents’ food decisions are the product of negotiation between independence, social context, and practical constraints. Interventions that target only one of these elements for example, health education without addressing cost or availability, are unlikely to have meaningful impact.

Marketing exposure was another strong driver. Branding, media, and packaging increased both attraction to and consumption of UPFs. This observation aligns with global evidence that advertising significantly shapes dietary preferences and intake among young people [42, 43]. Participants were divided on the idea of restricting such promotions: some feared a loss of useful information, while others welcomed stricter controls on unhealthy products. This mirrors broader policy debates, where voluntary industry self-regulation has been criticized as inadequate and international health agencies now advocate for comprehensive restrictions [44]. In this study, packaging appeared influential primarily through its association with hygiene, safety (e.g., intact wrapping and expiry dates), and visual appeal, rather than through detailed interpretation of nutritional labels.

Importantly, adolescents also offered thoughtful recommendations. They suggested checking product quality, limiting consumption, preferring natural foods, and strengthening regulations. Such reflections indicate that adolescents are not passive consumers but capable of critical engagement. Interventions should therefore move beyond individual education to address school food environments, regulate marketing, and influence social norms that encourage UPF consumption.

A key strength of this study is the use of systematic qualitative procedures, including iterative data engagement, peer debriefing, and maintenance of an audit trail, which enhanced the credibility and transparency of the findings. Although efforts were made to provide clear contextual descriptions, the findings remain context-specific and may not be fully transferable to other settings. Overall, this study provides context-specific evidence on the drivers and barriers to UPF use among adolescents in urban Ethiopia. The findings support multi-level interventions that combine education, environmental change, and regulatory measures, contributing to the global agenda to reduce premature deaths from NCDs, as outlined under United Nations SDG 3.4. More broadly, these findings are also relevant to SDG 2.2, which aims to end all forms of malnutrition, by highlighting the importance of healthier food environments and improved diet quality among adolescents. Although this qualitative study did not assess dietary diversity or directly contribute to SDG indicator 2.2.4 (minimum dietary diversity), it provides contextual evidence that may help inform policies promoting healthier adolescent diets. Moreover, these findings are consistent with global evidence highlighting the growing influence of UPF industries and marketing on children’s diets in LMICs, and the need for stronger regulatory action to create healthier food environments [45].

Furthermore, this study was limited by its small sample size and focus on a single urban setting, which may affect transferability to other contexts. As interviews relied on self-reported behaviours, responses may have been influenced by social desirability bias. Furthermore, the study did not undertake a formal comparative analysis by sex or school type, which may limit the ability to identify subgroup-specific differences. While the study explored adolescents’ perceptions of ultra-processed foods, it did not systematically assess their understanding of food classification according to the NOVA framework; therefore, participants’ descriptions of “processed” or “industrial” foods may not fully align with scientifically defined UPFs. In addition, some perceptions such as why adolescents consider certain foods healthy or nutritious and the sources of these beliefs were not explored in depth. Although packaging and marketing emerged as influential factors, the study did not examine specific elements (labeling or branding) in detail. Nevertheless, the findings provide valuable qualitative insights into adolescents’ perceptions and experiences regarding UPFs consumption in urban Ethiopia. Future research should track dietary patterns over time and include rural populations to capture a more comprehensive picture of adolescent food practices in Ethiopia.

## Conclusion

Adolescents in Adama occupy a middle ground: they frequently consume UPFs because such products are convenient, affordable, and normalized by packaging and marketing, yet they often simultaneously suspect harm and call for moderation and better regulation. This ambivalence suggests readiness for interventions that combine structural change (marketing restrictions, school food policies, availability of affordable healthy options) with education that leverages adolescents’ existing concerns and promotes practical alternatives.

Policy makers and program designers should consider multi-level strategies; regulate and restrict aggressive marketing of UPFs to young people, improve labeling and enforce safety/quality standards transparently, make healthier foods more accessible and affordable in school and neighborhood environments, and co-design education campaigns with adolescents so messages resonate with their lived priorities. Combining these elements may reduce reliance on UPFs while respecting adolescents’ need for convenience and autonomy. These recommendations align with recent global calls for stronger regulation of UPFs marketing and improved food environments to support healthier dietary behaviors among children and adolescents.

## Supplementary Information

Supplementary Material 1.

Supplementary Material 2.

Supplementary Material 3.

## Data Availability

Data from this study is available from the corresponding author upon request, subject to ethical restrictions. The interview guide and summary coding framework used in this study are provided as supplementary materials.
