# Supplementary material for: Ultra-processed food consumption, dietary behaviors, and marketing exposure among school adolescents in urban Ethiopia: a qualitative study
Source: BMC Nutr. 2026 Jun 15;12:178. doi: 10.1186/s40795-026-01401-5 (PMC13495184; doi:10.1186/s40795-026-01401-5)
Supplement: Supplementary file 1 [file 40795_2026_1401_MOESM2_ESM.docx]

**Guide questions**

1. What do you think are healthy foods? (probe to give examples, why did they say those food stuffs are healthy)
2. what about unhealthy foods? (probe to give examples, why did they say those food stuffs are unhealthy)
3. Have you ever heard about processed/ultra-processed foods? (probe for source of information, what do you know about those foods, what did you hear about those foods, types of foods/examples)
4. Can you describe what you usually eat and drink in a typical day? (What do you usually eat at school? At home? snacks or drinks do you usually have? frequency? Where do you usually get them from? (probe for their preference)
5. You mentioned foods like (participant’s own examples). Some of these may be packaged or ready-made foods What motivates you to eat these foods (UPFs)? (Probe for Cost, Flavor, Availability, Accessibility, comfort, health benefit etc.))
6. What is your intension to consume such foods? (Probe barriers and enablers for intention to use UPFs, including the influence of family, friends, relatives or other referents)
7. What do you think that the benefits of consumption of these UPF? (Probe for health, economic and social benefits)
8. What do you think are the risks of UPF consumption? (probe for safety related issues, its impact on physical, social, mental health . please provide examples)
9. What is your thinking or perception about UPFs? (probe for their inclination or judgement of those foods including the benefits, and harm aspects of using UPF, how frequent should be used)
10. Tell us about the foods you buy most often when shopping at [*the supermarket, suiq,*]. (Probe for reasons to buy those foods (why), like cost/affordability, availability or health aspect)
11. Tell us about the types of ready-to-eat meals you buy? (Probe for the reasons ‘ why or why not”)
12. What drives your decision making when choosing what foods to eat? (Probe for motivation, self-efficacy, autonomy, peer/family influence (social pressure), knowledge, attitude, perception etc)
13. What influenced you to buy UPFs? (Probe for advertisement, branding, access, health benefits, cost)
14. What do you think the influence of advertising and packaging on the decision to use healthy diets? (Probe for positive or negative effect of advertising UPFs, tiredness or hungery etc)
15. As an adolescent, what do you think the influence of not advertising UPFs on eating pattern? (probe for the likelihood of eating healthy/unhealthy diet, and reasons for choosing, what do you think about the issue restricting advertising unhealthy foods)
16. What do you recommend about the consumption of UPFs?
17. Is there anything that you would like to add?
