# Supplementary material for: Ultra-processed food consumption, dietary behaviors, and marketing exposure among school adolescents in urban Ethiopia: a qualitative study
Source: BMC Nutr. 2026 Jun 15;12:178. doi: 10.1186/s40795-026-01401-5 (PMC13495184; doi:10.1186/s40795-026-01401-5)
Supplement: Supplementary file 2 [file 40795_2026_1401_MOESM3_ESM.docx]

**Table 2: Theme and sub-themes**

| **S.N** | **Theme** | **Subtheme** |
| --- | --- | --- |
| 1 | Perceptions of UPFs | UPFs seen as clean/quality/tested |
|  |  | UPFs seen as affordable/accessible/time-saving |
|  |  | UPFs seen as unhealthy/artificial |
|  |  | Balanced/mixed perception |
| 2 | Intention to Consume | Autonomous choice (no pressure) |
|  |  | Social influence (family/peers) |
|  |  | Situational convenience (when no home food) |
|  |  | Economic enabler (cheaper than alternatives) |
|  |  | Taste/flavor preference |
|  |  | Availability/accessibility/location |
|  |  | Health-conscious choice |
| 3 | Consumption Behaviors | Preferred UPFs/snacks/sugary drinks |
|  |  | Staple convenience foods |
|  |  | Fruits/vegetables preference |
|  |  | Frequency high (≥4-5 days/week or daily) |
|  |  | Frequency moderate/low (≤3 days/week / occasionally) |
| 4 | Perceived Benefits | Nutritional/energy benefits |
|  |  | Safety/quality/standard benefits |
|  |  | Economic/social benefits |
| 5 | Perceived Risks | Health risks (teeth, obesity, organs) |
|  |  | Expired/packaging/contamination risks |
|  |  | Addiction/mental impact |
| 6 | Marketing Exposure & Effects | Advertising increases desire/uptake |
|  |  | Packaging/branding cues |
|  |  | Skepticism about misleading ads |
|  |  | Support for advertising bans or limits |
| 7 | Recommendation | Check expiry/quality before use |
|  |  | Moderation/limit frequency |
|  |  | Prefer natural/home-made foods |
|  |  | Improve regulation/education |
